# Fish Allergens at a Glance: Variable Allergenicity of Parvalbumins, the Major Fish Allergens

**DOI:** 10.3389/fimmu.2014.00179

**Published:** 2014-04-22

**Authors:** Annette Kuehn, Ines Swoboda, Karthik Arumugam, Christiane Hilger, François Hentges

**Affiliations:** ^1^Laboratory of Immunogenetics and Allergology, Public Research Centre for Health (CRP-Santé), Luxembourg, Luxembourg; ^2^Molecular Biotechnology Section, University of Applied Sciences, Vienna, Austria; ^3^Unit of Immunology and Allergology, Centre Hospitalier de Luxembourg, Luxembourg, Luxembourg

**Keywords:** allergenicity, fish allergy, fish gelatin, food allergy, isoallergens, isoforms, monosensitivity, parvalbumin

## Abstract

Fish is a common trigger of severe, food-allergic reactions. Only a limited number of proteins induce specific IgE-mediated immune reactions. The major fish allergens are the parvalbumins. They are members of the calcium-binding EF-hand protein family characterized by a conserved protein structure. They represent highly cross-reactive allergens for patients with specific IgE to conserved epitopes. These patients might experience clinical reactions with various fish species. On the other hand, some individuals have IgE antibodies directed against unique, species-specific parvalbumin epitopes, and these patients show clinical symptoms only with certain fish species. Furthermore, different parvalbumin isoforms and isoallergens are present in the same fish and might display variable allergenicity. This was shown for salmon homologs, where only a single parvalbumin (beta-1) isoform was identified as allergen in specific patients. In addition to the parvalbumins, several other fish proteins, enolases, aldolases, and fish gelatin, seem to be important allergens. New clinical and molecular insights advanced the knowledge and understanding of fish allergy in the last years. These findings were useful for the advancement of the IgE-based diagnosis and also for the management of fish allergies consisting of advice and treatment of fish-allergic patients.

## Fish Allergy

### Prevalence

In human diet, fish is a valuable source of essential amino acids, polyunsaturated fatty acids, and lipid-soluble vitamins. Although the fish consumption in European countries is quite stable, the global demand for fish and fish products is increasing still steadily (1). While many literature sources indicate that allergies to fish are on the rise, the actual prevalence is not well established as most studies are based on self-reported food allergies (2). It is estimated that up to 0.2% of the general population is affected by fish allergy (3, 4). However, the exposure to fish is an important factor determining the reported prevalence of fish allergy. Therefore, the prevalence of fish allergy is greater in countries with high fish consumption and fish-processing industries (5).

### Clinical features

Fish allergy is a pathophysiological, IgE-mediated immune response to specific fish proteins. Patients become sensitized by allergen exposure via the gastro-intestinal tract during ingestion, which is the major route of sensitization, or via the respiratory system by fish aeroallergens or skin contact (6–9). Common clinical manifestations are oral allergy syndrome, rhinitis, abdominal pain, diarrhea, urticaria, angioedema, asthma, and in severe cases, even life-threatening anaphylaxis (10–12). Fish aeroallergens can be important triggers of atopic dermatitis (13). There are only a few clinical studies addressing minimal eliciting doses of fish allergy. However, already low milligram amounts of fish seem to be sufficient to trigger allergic symptoms in sensitized patients (14). Cross-reactivity among fish species has been commonly reported for fish allergy (11, 15, 16). This clinical cross-reactivity seems to be even more pronounced between closely related fishes. On the other hand, it has been shown that even highly fish-sensitized patients can tolerate certain fish species, such as tuna (17, 18). In addition, in case reports, clinical monosensitivity to single fishes has been proven for sole, swordfish, pangasius/tilapia, tuna/marlin, and more recently, for salmon and salmonid fishes (19–26). So far, no cross-reactivity has been shown for fish and other seafood allergens while in some cases, a co-sensitization to both allergen sources might occur (27).

### Diagnosis and patient care

The diagnostic procedure is based on four main pillars: the patient’s clinical history, *in vivo* analysis of skin reactivity, *in vitro* quantification of specific serum IgE and, in selected cases, oral provocation challenges. A broad diversity of fishes is globally consumed but only a limited number of commercial extracts are available for skin testing. Therefore, fresh or processed fish is commonly used for this *in vivo* analysis. However, the predictive value of skin tests is low (28). For *in vitro* analysis of specific IgE levels, the ImmunoCAP system (ThermoScientific) offers a wide panel of fish extracts. Meanwhile, two recombinant parvalbumins from carp and cod are available for this diagnostic assay. The predictive value of fish extract-specific IgE measurements is not well established, but a high titer of specific IgE (20 kU_A_/L) was reported to predict an allergy to cod with a likelihood of 95% (29).

Other adverse reactions might be misdiagnosed as fish allergy (30). Allergy-like symptoms occur upon ingestion of histamine-contaminated, spoiled fish (“scombroid fish poisoning”) (31). Also, consumption of fish contaminated with the parasite *Anisakis simplex* (herring worm) provokes acute allergic manifestations caused by IgE-mediated sensitization to *Anisakis* allergens (27, 32).

To avoid severe reactions, the management of fish allergy relies on the elimination of each fish product from the diet of the sensitized patient. In some cases, it has been reported that patients may lose their sensitivity upon eliminating diet (33, 34). The therapeutic desensitization to fish has been reported only for a single case (35). The development of specific immunotherapeutics based on hypoallergenic variants of parvalbumins, the major fish allergens, is the focus of ongoing studies (36, 37). Research on a primary strategy for the prevention of fish allergy is very limited. The current recommendations of the American Academy of Allergy, Asthma & Immunology also do not suggest a general, delayed introduction of fish in the diet of children (38).

## Fish Allergens

### Food allergens

The few foods that are responsible for causing most allergic reactions are milk, eggs, peanuts, tree nuts, fish, shellfish, soy, and wheat. They contain potent food allergens (39). Their allergenic potency has been related to specific protein features. These allergens are highly abundant in the food sources, and moreover, they possess a high stability toward food processing and digestion (40). The structural stability has been allocated to different protein characteristics such as intrinsic ligand binding and intramolecular disulfide bonds (41). Some food allergens form protein aggregates of high stability. Although some food allergens are sensitive to gastric and intestinal digestion, degradation fragments are still recognized by specific IgE antibodies (42). Food allergens of animal origin are mainly grouped into three protein superfamilies such as caseins, tropomyosins, and EF-hand proteins (43).

### Fish parvalbumins

Most fish parvalbumins belong to the beta-subtype while the alpha-subtype is predominantly found in other organisms. Beta-parvalbumin has first been identified as fish allergen in Baltic cod (44). Later on, the importance of this protein as the fish panallergen was confirmed for a wide range of commonly consumed species such as salmon, carp, mackerel, tuna, and pilchard (45–49). Parvalbumins are highly stable, low-molecular-weight proteins (10–12 kDa), which are very common in fish muscle (5). The muscle of bony fishes is composed of two tissues, the light and dark muscle differing by their physiological function and composition (50). The parvalbumin expression is considerably higher in light than in dark muscle tissue (51). In contrast to most bony fishes, pelagic fishes such as tuna have mainly dark muscles of low parvalbumin content (48). This complies with the low allergenicity reported for tuna which is even used in canned preparations as matrix for food challenges (17). Overall, the parvalbumin content differs considerably in fish species and the different amount of parvalbumins correlates with the variable allergenicity of fishes (52–54). It has been shown that the parvalbumin level is up to 100-times higher in carp than in mackerel or tuna muscle. In addition, physical and chemical effects of food processing may alter the allergenicity of fish in food preparations by parvalbumin degradation or oligomerization which may decrease or increase the number of IgE epitopes (55).

Parvalbumins belong to the family of EF-hand proteins. As such, they contain specific EF-hand motifs composed of 12 residues of long loops which are involved in the binding of divalent metal ions. The N-terminal EF-hand site is a non-functional domain while the two other EF-hand motifs are binding calcium and magnesium ions (56). The physiological role of muscle parvalbumins is related to the regulation of the intracellular calcium concentration during muscle relaxation (57). Upon ion binding and ion release, the beta-parvalbumin structure is subjected to a global rearrangement indicating a general flexibility of the EF-hand domains (58). The apo-protein, which is calcium-depleted parvalbumin, has a reduced ability to bind IgE antibodies from fish-allergic patients (59). Consequently, these calcium-binding protein regions were attributed to conformational B-cell epitopes (60).

As of today, the official database of allergens contains 21 parvalbumins from 12 fish species^1^ (Table 1) (61). The Allergome database lists far more than 100 entries for fish parvalbumins and their isoforms/isoallergens^2^ (62). This much higher number arises from a compilation of homolog, potentially allergenic molecules, in addition to proteins of proven allergenicity. Parvalbumin has been defined as the major fish allergen as a majority of fish-allergic patients have IgE antibodies reacting to this muscle protein (63–65). However, the prevalence of parvalbumin-specific IgE antibodies seems to vary across different patient populations. For example, fish-allergic patients with sensitization to tropical fishes react mostly to allergens other than parvalbumins (66, 67). Generally, the high clinical cross-reactivity among fishes has been attributed to cross-reacting IgE antibodies recognizing parvalbumins from several species (68). Recently, clinical monosensitivity to salmonid fishes has been linked to salmonid parvalbumin-specific IgE antibodies suggesting that cross-reactivity among fish parvalbumins may be restricted to these closely related fishes (23, 25).

**Table 1:** **Entries of official fish allergens by the International Union of Immunological Societies Allergen Nomenclature Subcommittee database (www.allergen.org)**.

Order	Fish	Allergen name	Protein identity
Clupeiformes	*Clupea harengus* (Atlantic herring)	Clu h 1.0101	Parvalbumin
		Clu h 1.0201
		Clu h 1.0301
	*Sardinops sagax* (Pacific pilchard)	Sar sa 1.0101	Parvalbumin
Cypriniformes	*Cyprinus carpio* (common carp)	Cyp c 1.0101	Parvalbumin
		Cyp c 1.0201
Gadiformes	*Gadus callarias* (Baltic cod)	Gad c 1.0101	Parvalbumin
		Gad m 1.0101	Parvalbumin
		Gad m 1.0102	
		Gad m 1.0201	
	*Gadus morhua* (Atlantic cod)	Gad m 1.0202	
		Gad m 2.0101	Enolase
		Gad m 3.0101	Aldolase
Perciformes	*Lates calcarifer* (barramundi)	Lat c 1.0101	Parvalbumin
		Lat c 1.0201	
	*Oreochromis mossambicus* (tilapia)	Ore m 4.0101	Tropomyosin
	*Thunnus albacares* (yellowfin tuna)	Thu a 1.0101	Parvalbumin
		Thu a 2.0101	Enolase
		Thu a 3.0101	Aldolase
	*Xiphias gladius* (swordfish)	Xip g 1.0101	Parvalbumin
Pleuronectiformes	*Lepidorhombus whiffiagonis* (megrim)	Lep w 1.0101	Parvalbumin
Salmoniformes	*Oncorhynchus keta* (Pacific salmon)	Onc k 5.0101	Vitellogenin
	*Oncorhynchus mykiss* (rainbow trout)	Onc m 1.0101Onc m 1.0201	Parvalbumin
	*Salmo salar* (Atlantic salmon)	Sal s 1.0101	Parvalbumin
		Sal s 2.0101	Enolase
		Sal s 3.0101	Aldolase
Scorpaeniformes	*Sebastes marinus* (redfish)	Seb m 1.0101	Parvalbumin
		Seb m 1.0201	

### Fish enolases and aldolases

Initially, IgE reactivity to fish beta-enolase and aldolase had been described in single fishes (69–71). In 2013, 50 kDa enolases and 40 kDa aldolases were identified as important fish allergens in cod, salmon, and tuna (Table 1) (72). Both enzymes are abundant in the fish muscle as they are involved in metabolic glycolysis, the sugar degradation during physiological production of energy.

In a recent study, IgE binding to enolases and aldolases was detected in a high number of fish-allergic patients (72). Most of the enolase/aldolase-positive patients also had IgE antibodies to parvalbumin. Both allergens were positive in the mediator release assay using rat basophilic leukemia cells expressing the human high-affinity IgE receptor passively sensitized with patient sera. They were found to be sensitive to heat treatment. Their relevance as food allergens is still not well understood especially in parvalbumin-negative patients.

### Fish gelatin

The relevance of fish gelatin as a food allergen has been controversially discussed for many years (4). Fish gelatin is commonly used in food and pharmaceutical products replacing mammalian gelatins (73).

Originally, IgE binding to fish collagen had been shown in fish-allergic patients (74, 75). Later, the prevalence of fish gelatin allergy was addressed in single clinical studies (76, 77). In one study, skin prick tests were positive in 3 of 30 cod-allergic patients, while food challenges were positive in one of the three tested patients. Also, its potency as a food allergen was shown in a case report of severe anaphylaxis upon ingestion of sweets containing several grams of fish gelatin (78). No cross-reactivity of mammalian and fish gelatin has been reported so far. Another risk factor of fish gelatin is a potential contamination with fish parvalbumin as it is produced from fish as a natural source. In fact, parvalbumin traces were detected in isinglass, a fish collagen-based food supplement (79).

### Other IgE-reactive fish proteins

In the past decades, numerous studies reported allergens different from parvalbumin which were specified by their molecular weight or identified by other methods. Although most studies showed IgE reactivity to these proteins, the relevance of these potential allergens was not addressed. Several allergens (63 kDa protein, further IgE-reactive proteins) were described for cod while some were identified as parvalbumin oligomers (80). Furthermore, allergens were identified in swordfish (25 kDa), eel/eelpout (40 kDa), snapper (35–90 kDa), tuna/marlin (94–105 kDa), scad (46–50 kDa), tropical fishes (29–54 kDa), and pangasius/tilapia (18–45 kDa) (20–22, 64, 66, 81, 82). Potential allergens of known identity were found in cod (aldehyde phosphate isomerase), salmon (triose-phosphate isomerase, fructose-bis-phosphate isomerase, serum albumin), and tuna (creatine kinase, beta-enolase) (70, 83, 84).

Two further fish allergens have been registered by the IUIS-allergen database (Table 1). Food allergy to fish roe has been addressed in a few studies so far. Vitellogenin, a fish yolk protein, has been identified as a food allergen in caviar from different fishes (85, 86). In 2013, tropomyosin, a filamentous muscle protein, was purified, cloned, and identified as a fish allergen in tilapia-sensitized patients (87). This tilapia allergen showed high homology (88%) to the human homolog. It was suggested that it may be involved in autoimmune reaction of inflammatory bowel disease as a significant proportion of the patients with IgE to tropomyosin suffered from this autoimmune disease.

## Allergenicity of Parvalbumins

During last decades, parvalbumins, enolases, aldolases, and fish gelatin have been identified as allergenic fish proteins. Specific properties have been attributed to common food allergens such as resistance toward conditions of the gastrointestinal tract and influences by food processing conditions (88). So far, only the allergenicity of parvalbumins has been characterized in more detail as to their allergenicity while consolidated findings still need to be generated for the other allergens.

### Parvalbumins of the alpha- and beta-lineage

Parvalbumins have been classified into two phylogenetic lineages, alpha and beta subtype. Their isoelectric points (alpha, pI > 5.0; beta, pI < 4.5) and the other multiple characteristics of the primary protein structure are different (89, 90). Generally, parvalbumins occur in various organs such as the central nervous system and the muscles (56). In muscles, both subtypes have been detected in amphibian tissues while only the alpha-subtype has been reported in mammalian and avian muscles (91, 92). Cartilaginous fish muscles express alpha-parvalbumins whereas the beta-homolog is found in muscle tissue of bony fishes (93). So far, the potency as important food allergens has been shown only for fish beta-parvalbumins. Despite their overall structural similarity to beta-homologs, alpha-parvalbumins are generally considered as non-allergenic proteins (43). Indeed, parvalbumins of the alpha-lineage have been described only as frog meat allergens (94). However, IgE cross-reactivity has also been reported between homologs from fish and frog in a population of fish-sensitized patients (95). In 2011, chicken alpha-parvalbumin has been reported as a poultry allergen in chicken meat allergy (96). Interestingly, IgE recognition of both fish and avian homologs was found in one case study while cross-reactivity was not detected in another clinical case (97, 98). However, the sequence identity between fish parvalbumins and homologs from mammals and birds is low (<55%) so that IgE cross-reactivity seems to be unlikely.

Recent classification by structural, biochemical, and phylogenetic analysis assigned alpha-parvalbumins into a common cluster together with higher vertebrate, including human proteins while beta-subtypes form a separate, only remotely related cluster of parvalbumins which might explain the variable allergenicity of both lineages (99, 100).

### Antigenic determinants of parvalbumin

The amino acid sequence identities of fish parvalbumins vary substantially (55–95%) but high structural similarity has been reported as a common protein characteristic (101). IgE-binding epitopes were suggested to be located in highly conserved, calcium-binding regions of the molecule. Indeed, reduced IgE binding was found for parvalbumin mutants with single amino acid modifications in calcium-binding motifs (60). The analysis of human B-cell epitopes has only been addressed so far in a few parvalbumins namely allergens from cod, carp, mackerel, and salmon (25, 102–104).

Both linear and conformational epitopes were found in these studies but overall, non-plausible peptide pattern was found to define a common IgE-binding region of fish parvalbumins. It seems that B-cell epitopes are distributed over the whole parvalbumin primary structure. A possible explanation might be the polyclonal B-cell response in fish-allergic patients, which is even more stimulated by individual’s eating habits (e.g., canned, fried, smoked fish) leading to a high variety of exposed allergen forms such as native, modified, or degraded parvalbumins from different species.

### Parvalbumin isoforms of variable allergenicity

For most patients, clinical cross-reactivity among fish species was postulated as a specific feature of fish allergy. Although single clinical reports showed true monosensitivity to single fishes, there was no allergen-based explanation for these observations. Only recently, the species-specific sensitization was attributed to the presence of IgE antibodies selectively recognizing a single salmonid parvalbumin isoallergen (beta-1 parvalbumin) (25). These findings may allow two important conclusions concerning the allergenicity of parvalbumins.

First, parvalbumins from different fishes might vary by their allergenic potential. This might be explained by the specific sensitization of the individual patient, which results from the clinical history (eating habits, age of onset of fish allergy).

**Figure 1:**
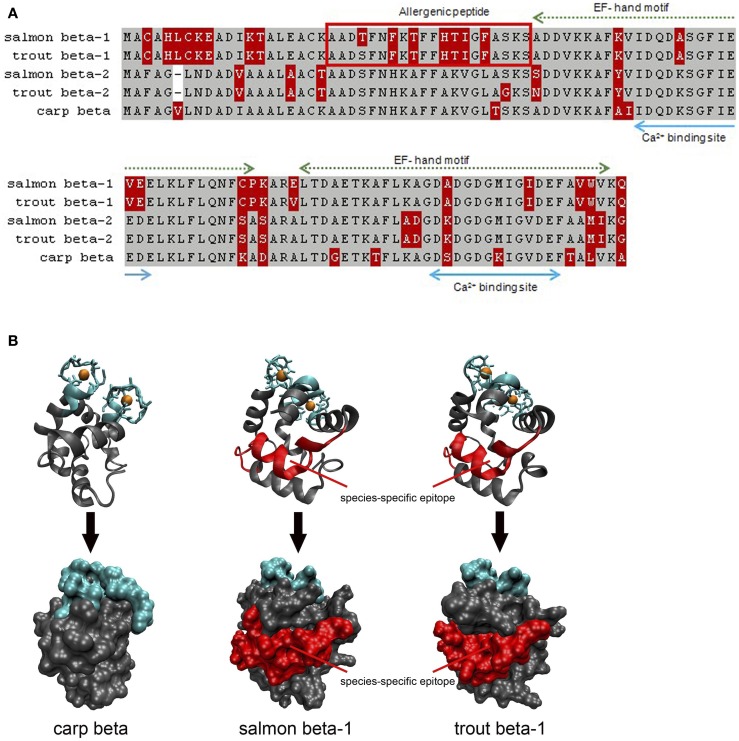
**Comparison of salmon (X97824, X97825), trout (FN544258, FN544259), and carp (P02618; 4cpv) parvalbumins**. **(A)** Both salmonid beta-1 parvalbumins sequences differ from their homologs in the N-terminal third of the protein which is not involved in calcium binding. The allergenic peptide is specifically recognized by IgE from a patient monosensitized to salmonid fishes. Gray, identical residues; red, variable residues. **(B)** Both Ribbon and surface models show that the allergenic peptide is localized on the surface of salmon and trout beta-1 parvalbumins. Blue, calcium-binding regions; red, IgE epitope.

Second, the allergenicity of parvalbumin isoforms/isoallergens from the same fish might be variable, as reported for salmon allergens. In fact, fishes express often a high number of parvalbumins such as that reported for carp and catfish (105, 106). These isoforms seem to play a physiological role in the fish muscle adaptation to developmental and environmental changes. In the same fish, parvalbumins differing by sequence microheterogeneity (sequence identity >90%) have been reported (99). It is conceivable that these highly identical isoforms might be of variable allergenicity. For Bet v 1, the major birch pollen allergen, a number of isoforms with different allergenic properties were isolated (107). Several allergenic parvalbumins have been identified in commonly consumed fish, cod (beta-1, beta-2), salmon (beta-1, beta-2), and herring (beta-1, beta-2, beta-3) (5). During studies of monosensitivity to salmonid fishes, different antigenic regions were assumed but only a single epitope was defined as a species-specific allergy marker (23, 25). This unique epitope was localized on a single isoform, the beta-1 salmon parvalbumin. For a patient with documented monosensitivity to salmonid fishes, we could confirm a single region from salmon and trout as species-specific parvalbumin epitope (“allergenic peptide”; Figure 1). This antigenic region was located on the parvalbumin surface and was unique when compared with different homologs from other fish species. The allergenic region matched with the IgE-binding epitope identified by a subsequent study using a peptide-based microarray assay (25). Studies with further sera from patients with specific sensitizations are required to demonstrate the existence of antigenic parvalbumin regions linked to specific clinical phenotypes. A recent study showed that 9 out of 62 fish-allergic patients (15%) experienced clinical reactions with salmonid fishes only (72). This suggests the conclusions that the prevalence of clinically salmon-monosensitized patients is higher than previously assumed.

## Conclusion

During recent decades, important insights into clinical and allergen-based features were gained for fish allergy: the variable allergenicity among salmon parvalbumins (beta-1, beta-2) was shown, in addition, important new fish allergens (enolase, aldolase, fish gelatin) were identified. These findings will help to develop new immunotherapeutic strategies, but they have also shown that the clinical picture of fish allergy is more complex than anticipated. New molecules need to be implemented in IgE-based routine assays to advance patient diagnosis and advice.

## Author Contributions

All authors have contributed to the conception, design, and drafting of the paper.

## Conflict of Interest Statement

The authors declare that the research was conducted in the absence of any commercial or financial relationships that could be construed as a potential conflict of interest.

## Footnotes

^1^www.allergen.org

^2^www.allergome.org
